# Reversible sensory neuropathy in mitochondrial trifunctional protein deficiency

**DOI:** 10.1002/jmd2.12279

**Published:** 2022-03-09

**Authors:** Sarah Catharina Grünert, Matthias Eckenweiler, Ute Spiekerkoetter

**Affiliations:** ^1^ Department of General Pediatrics, Adolescent Medicine and Neonatology, University Medical Center, Faculty of Medicine University of Freiburg Freiburg Germany; ^2^ Department of Neuropediatrics and Muscle Disorders, University Medical Center, Faculty of Medicine University of Freiburg Freiburg Germany

**Keywords:** electrophysiology, LCHAD, long‐chain 3‐hydroxyacyl‐CoA dehydrogenase deficiency, mitochondrial trifunctional protein deficiency, MTP, nerve conduction, neuropathy

## Abstract

Axonal peripheral neuropathy is a common complication of mitochondrial trifunctional protein (MTP) deficiency and long‐chain 3‐hydroxyacyl‐CoA dehydrogenase deficiency that is usually considered progressive. Current treatment strategies are not able to fully prevent neuropathic symptoms in the majority of patients. We herein report three sisters with genetically proven MTP deficiency who were untreated until adolescence, when electrophysiological studies first revealed isolated axonal sensory neuropathy. Apart from mild exercise intolerance and missing deep tendon reflexes of the lower extremities, all three girls were clinically asymptomatic. A fat‐reduced and fat‐modified diet together with a reduction of the nocturnal fasting time resulted in complete normalisation of the electrophysiological studies after 1 year of dietary treatment. Our findings suggest that neuropathy might be responsive to dietary interventions in MTP patients at a very early stage of disease.

**Synopsis**

Axonal peripheral neuropathy in MTP deficiency may be responsive to dietary treatment at an early stage of the disease.

## 1 INTRODUCTION

Peripheral neuropathy is a common complication of mitochondrial trifunctional protein (MTP; OMIM #609015) and long‐chain 3‐hydroxyacyl‐CoA dehydrogenase (LCHAD) deficiency (OMIM #609016) that has been reported to occur in up to 80% of MTP patients.^1^, ^2^ The pathophysiology of neuropathy in these disorders of long chain fatty acid oxidation is still not fully understood. Current treatment strategies such as the dietary restriction of long chain fatty acids and supplementation of medium chain triglycerides (MCTs) are not able to fully prevent neuropathic symptoms in the majority of patients.^1^, ^3^ The neuropathic phenotype is heterogeneous comprising isolated sensory,^4^, ^5^ isolated motor^1^ and sensorimotor forms.^1^ Neuropathy is usually considered irreversible.^3^ We recently outlined the clinical spectrum of peripheral neuropathy in a cohort of 8 LCHAD deficient and 11 MTP‐deficient patients.^1^ A novel observation in this cohort was that in patients with acute, fulminant onset of neuropathy, the neuropathic symptoms were at least partly reversible. All these patients had distinct neuropathic symptoms, mostly with loss of the ability to walk. In this work, we describe three MTP‐deficient sisters with beginning isolated sensory neuropathy that was completely reversible under dietary treatment. Our findings suggest that dietary interventions might improve neuropathy at an early disease stage.

## 2 CASE PRESENTATIONS

Clinical data of all three patients have been previously reported in the original study.^1^ Electrophysiological studies were performed as described.^1^

### 2.1 Patient 1

Patient 1 is the oldest child of the family showing a mild, neuropathic phenotype. Her newborn screening result was suggestive of multiple acyl‐CoA dehydrogenase deficiency [elevated concentrations of C6, C8 (0.24 μmol/L, normal <0.2 μmol/L), C10, C10:1, C12, C12:1, C14:1 (1.3 μmol/L, normal <0.36 μmol/L) and C14:2, as well as glutarylcarnitine (0.26 μmol/L, normal <0.13 μmol/L)], but repeated acylcarnitine analyses in dried blood and urine organic acid analysis yielded normal results. She presented with an ataxic gait, a tendency to stumble and with problems climbing stairs and getting up from squatting at the age of 4 years. All these symptoms were triggered by a febrile infection. Deep tendon reflexes of the lower extremities were absent. Surprisingly, these neurologic symptoms subsided within a few months and her further development was normal. The diagnosis was made at the age of 6 years, when she had a first mild metabolic decompensation triggered by febrile gastroenteritis. Due to a suggestive acylcarnitine result, genetic analysis of the *HADHA* gene was performed and yielded compound heterozygosity for two gene variants, c.1828C>G, p.(R610G) and c.2281T>G, p.(F761V). No further metabolic decompensations occurred, and apart from mild exercise intolerance the girl remained asymptomatic. At the age of 11.5 years, electrophysiological studies were performed as part of the routine follow‐up and revealed an isolated axonal sensory neuropathy. Deep tendon reflexes of the lower extremities were absent. The patient additionally presented first signs of retinopathy on the left side. Therefore, a fat‐restricted and fat‐modified diet was initiated (total fat 25%–30% of total energy, thereof about 40% MCTs), and uncooked cornstarch (0.75 g/kg body weight) was supplemented at bedtime to reduce fasting time. Under this regimen, exercise tolerance improved. Electrophysiological follow‐up studies after 1 year of dietary treatment showed complete normalisation of the previously impaired sensory amplitudes and beyond that a marked rise in the motoric amplitudes. The retinopathy remained stable.

### 2.2 Patients 2 and 3

**FIGURE 1:**
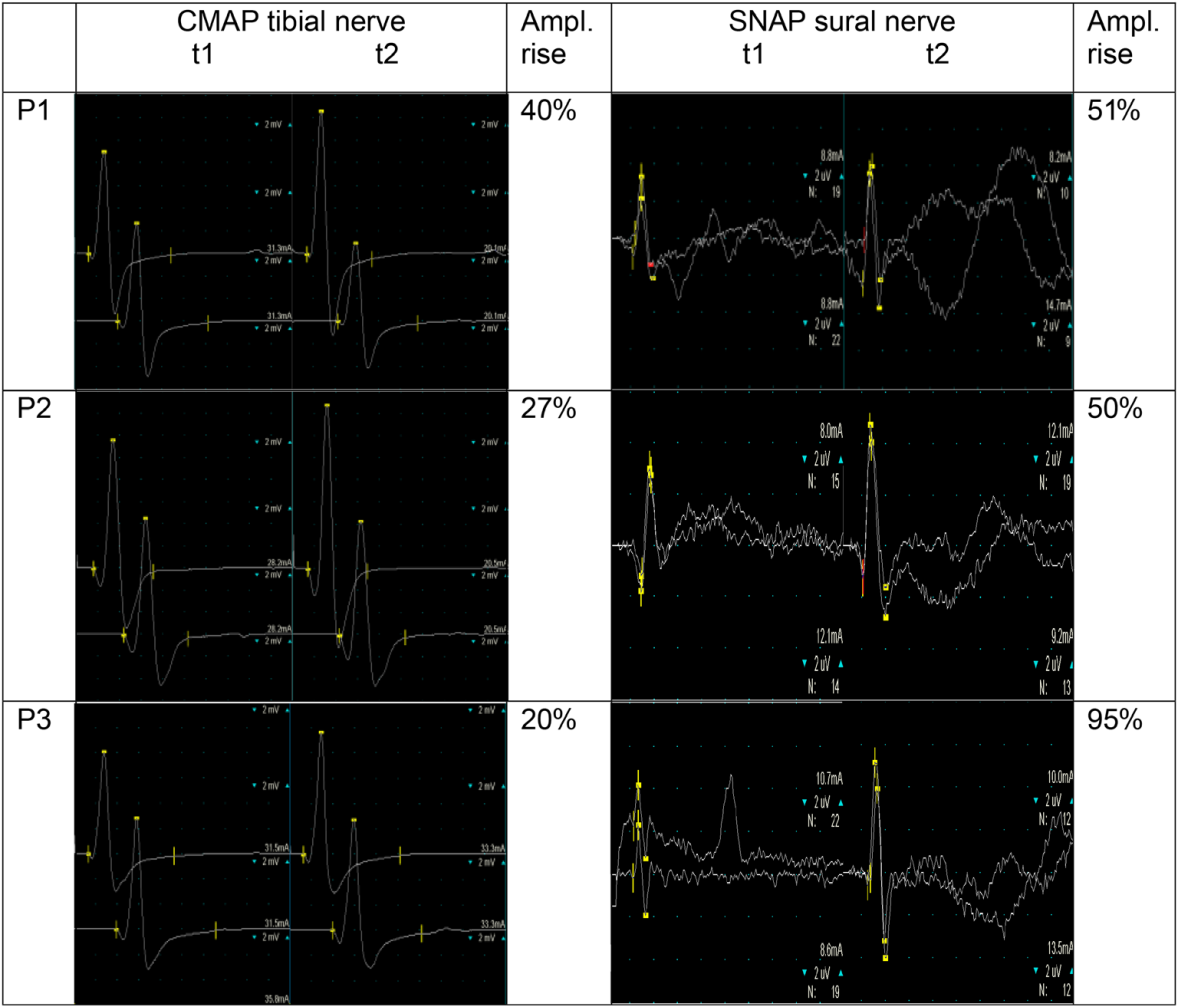
Compound muscle action potential of the left tibial nerve and sensory nerve action potential of the right sural nerve of patient 1–3 before dietary treatment (t1) and after 12 months of dietary treatment (t2) showing a distinct increase in both the motoric and sensory amplitude. Ampl, amplitude; CMAP, compound muscle action potential; P1/P2/P3, patient 1/2/3; SNAP, sensory nerve action potential; t1, time of study before starting adapted diet; t2, time of study 12 months after starting adapted diet

Patients 2 and 3 are the younger twin sisters of patient 1. Newborn screening results were unremarkable in both children. Apart from mild exercise intolerance, they have remained asymptomatic so far. They were diagnosed with MTP deficiency at the age of 5 years following the genetic diagnosis of their older sister. Both patients were found to harbour the same genetic *HADHA* variants. Due to the lack of clinical symptoms, no dietary treatment was initiated at that time. Similar to their older sister, nerve conduction studies at the age of 9 years revealed isolated axonal sensory neuropathy, and deep tendon reflexes of the lower extremities were absent. Dietary treatment was initiated as mentioned above and again resulted in improvement of exercise intolerance and complete normalisation of electrophysiological studies after 1 year of dietary treatment (Figure 1).

## 3 DISCUSSION

Axonal peripheral neuropathy is a common long‐term complication of MTP deficiency that affects the majority of patients and often occurs already during childhood.^1^ While until recently, neuropathy was considered irreversible and progressive,^3^ we have reported several individuals with LCHAD or MTP deficiency in whom neuropathic symptoms at least partially resolved.^1^ However, reversibility of neuropathic symptoms has so far only been observed in patients with acute onset or aggravation of neuropathy following febrile infections or catabolic episodes. Our new findings suggest that neuronal damage might also be improved by dietary interventions in patients at an early stage of disease. The underlying pathophysiology of the mainly axonal damage in MTP deficiency is still not fully understood. Toxicity of accumulating hydroxyacyl species has been suggested to contribute to the pathophysiology.^6^, ^7^, ^8^ In our patients, the concentrations of long‐chain 3‐hydroxyacylcarnitines in blood before start of dietary treatment were in the low normal range. Although we could not detect a significant decrease of these potentially toxic metabolites under the fat‐reduced diet in blood (mainly due to the already very low concentrations of these metabolites at baseline) we hypothesise, that the dietary modifications might have contributed to the reversibility of neuropathy. It has previously been reported that blood and tissue concentrations of carnitine and acylcarnitines do not correlate, therefore we speculate that dietary intervention may have resulted in a reduction of hydroxyacyl compounds at tissue level. Given the role of the LCHAD enzyme in the biosynthesis of cardiolipin, cardiolipin deficiency with secondary mitochondrial respiratory chain dysfunction has also been discussed as a potential pathophysiological mechanism of LCHAD/MTP neuropathy and also retinopathy.

If the positive effects of dietary intervention in our patients are long‐lasting, remains to be observed. Responsiveness to treatment may also depend on the genotype, and it is possible that one or both of the genetic variants identified in this family are more responsive to dietary treatment than others.

## 4 CONCLUSIONS

Our findings suggest that neuropathy may be responsive to dietary interventions at an early stage of the disease. Further studies are needed to elucidate the pathophysiological factors contributing to the axonal damage and identify possible treatment targets.

## CONFLICT OF INTEREST

The authors declare that they have no conflict of interests.

## AUTHOR CONTRIBUTIONS

All authors were involved in the clinical management of the patients. S. C Grünert drafted the manuscript, and M. Eckenweiler drafted Figure 1. M. Eckenweiler and U. Spiekerkoetter critically revised the manuscript. All authors approved the final version of the manuscript.

## ETHICS APPROVAL

Ethics approval is not applicable for this article.

## CONSENT FOR PUBLICATION

The patients' parents gave their informed consent for the publication of this case report.

## Data Availability

Data sharing is not applicable to this article as no new data were created or analysed in this study.
